# Cycling and activated CD8^+^ T lymphocytes and their association with disease severity in influenza patients

**DOI:** 10.1186/s12865-022-00516-1

**Published:** 2022-09-05

**Authors:** Shuai Liu, Zhisheng Huang, Ruyue Fan, Ju Jia, Xiaoyan Deng, Xiaohui Zou, Hui Li, Bin Cao

**Affiliations:** 1 Department of Respiratory and Critical Care Medicine, Shandong Provincial Hospital Affiliated to Shandong First Medical University, Jinan, Shandong China; 2 Shandong Key Laboratory of Infectious Respiratory Disease, Jinan, Shandong China; 3 Institute of Respiratory Medicine, Chinese Academy of Medical Sciences, Peking Union Medical College, Beijing, China; 4 Department of Pulmonary and Critical Care Medicine, Center for Respiratory Diseases, China-Japan Friendship Hospital, Beijing, China; 5 Shandong Center for Disease Control and Prevention, Jinan, Shandong China; 6 Tsinghua University-Peking University Joint Center for Life Sciences, Tsinghua University, Beijing, China

**Keywords:** Severe influenza infection, T cell function, Cell cycle

## Abstract

**Background:**

T cell lymphopenia was a significant characteristic of severe influenza infection and it was associated with the functional changes of T cells. It is necessary to clarify the T cells characteristics of kinetic changes and their correlation with disease severity.

**Methods:**

In a cohort of hospitalized influenza patients with varying degrees of severity, we characterized lymphocyte populations using flow cytometry.

**Results:**

The numbers of cycling (Ki67^+^) T cells at the acute phase of severe influenza were higher, especially in the memory (CD45RO^+^) T cell subsets. T cells from hospitalized influenza patients also had significantly higher levels of the exhausted marker PD-1. Cycling status of T cells was associated with T cell activation during the acute phase of influenza infection. The recruitment of cycling and activated (CD38^+^HLA-DR^+^) CD8^+^ T cells subset is delayed in severe influenza patients.

**Conclusions:**

The increased numbers of cycling memory (Ki67^+^CD45RO^+^) T cells subsets and delayed kinetics of activated (CD38^+^HLA-DR^+^) CD8^+^ T cells, could serve as possible biological markers for disease severity.

**Supplementary Information:**

The online version contains supplementary material available at 10.1186/s12865-022-00516-1.

## Introduction

Influenza viruses pose a persistent threat to global human health [1]. The degree of respiratory compromise caused by influenza viruses infection varies in different populations. Several viral, host and environmental factors impact disease severity leading to increased hospitalization and mortality [2]. Some studies identified that immune response, neutrophil function and cell cycle, as three distinct pathogenic mechanisms, were linked to disease progression of influenza infection [3–5]. T lymphocyte responses correlated with protection following influenza infection could be a sign to predict the disease severity [6]. Our previous clinical study revealed that T cell lymphopenia was a significant characteristic for severe influenza infection and reliable indicator in evaluating disease status [7, 8]. Although destruction of T lymphocytes by influenza viral particles undoubtedly led to cellular depletion in circulation [9, 10], other mechanisms, such as T cell excessive expansion-induced cellular turnover, have been implicated as well [11, 12]. However, the degree to which immune activation and cell cycle change is an underlying driver of T cell lymphopenia and even disease progression is not clear. Therefore, a cohesive understanding of the T cells function, especially cell cycle characteristics in influenza infection, could serve as markers for disease progression or severity.

Here, our objective was to identify specific immune signatures associated with severe influenza infection and the relationship among the phenotype or functionality of T cells. To this end, we comprehensively analyzed the clinical data and samples from 61 hospitalized patients of influenza infection. The kinetics of immune phenotype of T lymphocyte subset were longitudinally characterized. Our results thus provided a preliminary demonstration of cycling and activated CD8^+^ T cells had a delayed occurrence in severe patients.

## Results

### Clinical cohorts

Influenza patients were recruited in 2017–2018 and 2019–2020 (*n* = 61). Out of our total cohort of 61 subjects, 34 had “Moderate” and 27 as “Severe” disease according to criteria described in materials and methods. They were similar in terms of gender, age and infecting virus strains. All influenza patients enrolled had at least one comorbidity, with the most common being pneumonia. 84% patients (51 of 61) required supplemental oxygen. All patients with severe disease were admitted to the intensive care unit (ICU), and 44% patients (12 of 27) required Extracorporeal membrane oxygenation (ECMO). The hospital mortality rate in the severe cases of influenza pneumonitis were 52% (14 of 27). Cohort characteristics were listed in Table 1.

**Table 1:** Demographics and clinical characteristics of influenza patients

	Total (N = 61)	Moderate (N = 34)	Severe (N = 27)	P value
Gender				0.4904
Female	29 (48%)	18 (53%)	11 (41%)	
Male	32 (52%)	16 (47%)	16 (59%)	
Age/years (mean (SD))	55.68 (14.71)	59.00 (11.23)	51.51 (17.51)	0.0607
*Flu virus strains*				
Influenza type A	41 (67%)	25 (74%)	16 (59%)	0.1195
Influenza type B	18(30%)	7(20%)	11(41%)	
Influenza type A & B	2 (3%)	2 (6%)	0 (0%)	
*Comorbidity*				
Pneumonia disease	50 (82%)	23 (67%)	27 (100%)	0.0007
Cancer	3 (5%)	3 (8%)	0 (0%)	0.2475
Hypertension	19 (31%)	12 (35%)	7 (26%)	0.6125
Diabetes	14 (23%)	6 (17%)	8 (30%)	0.4243
*Laboratory tests (mean (SD))*				
Total leukocytes (× 10^9^/L)	9.96 (7.84)	7.26 (5.28)	12.66 (9.07)	0.0012
Neutrophil (× 10^9^/L)	8.34 (7.49)	5.51 (4.91)	11.18 (8.58)	0.0000
Proportion of Neutrophil (%)	79.15 (12.61)	71.77 (12.82)	86.53 (6.84)	0.0000
Lymphocytes (× 10^9^/L)	1.00 (0.56)	1.04 (0.55)	0.96 (0.57)	0.4585
Proportion of Lymphocytes (%)	13.14 (9.26)	17.42 (10.40)	8.86 (5.35)	0.0003
*Respiratory support*				
Extracorporeal membrane oxygenation	12 (20%)	0 (0%)	12 (44%)	0.0000
Invasive mechanical ventilation	27 (44%)	0 (0%)	27 (100%)	0.0000
Noninvasive mechanical ventilatory support	29 (48%)	16 (47%)	13 (48%)	1.0000
Require supplemental oxygen	51 (84%)	24 (71%)	27 (100%)	0.0015
*Outcome*				
Hospitalization	61 (100%)	34 (100%)	27 (100%)	0.3701
Admission to ICU	35 (57%)	9 (26%)	26 (96%)	0.0000
Death	14 (23%)	0 (0%)	14 (52%)	0.0000

Data are mean (SD) or n (%). p values were calculated by Student's t test, Mann–Whitney U test, χ2 test, or Fisher's exact test, as appropriate

### Increased memory CD8^+^ and CD4^+^ T cells cycling in severe patients during the acute phase of influenza infection

**Fig. 1:**
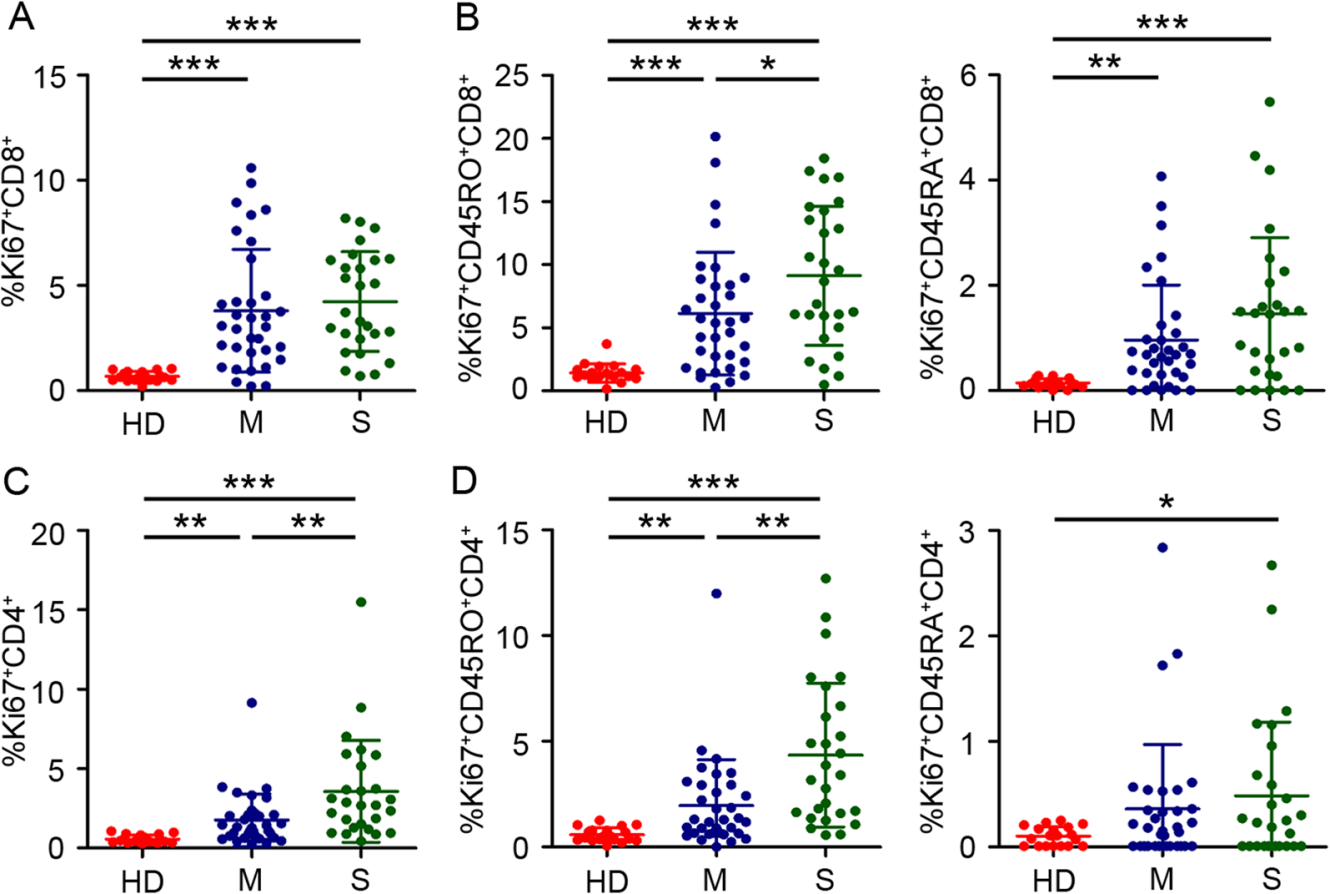
The percentage of cycling memory CD8^+^ and CD4^+^ T cells increased with disease severity of influenza infection. **A, B** Percentages of cycling CD8^+^ T cells, cycling CD8^+^ memory and effector T cells in healthy donors (HD, *n* = 19), moderate patients (M, *n* = 34) and severe patients (S, *n* = 27). **C, D** Percentages of cycling CD4^+^ T cells, cycling CD4^+^ memory and effector T cells in healthy donors and influenza patients. Data are displayed as mean ± SEM. Statistical significance is determined by unpaired t test. *P < 0.05, **P < 0.01, ***P < 0.001

We evaluated cycling T cell population using the intracellular expression of Ki67, a marker of ongoing or recent cell cycle entry[13, 14]. The gated strategy was shown in Additional file 1: Fig. S1B. We found that the percentage of cycling CD8^+^ T cells increased in patients during the acute phase of influenza infection compared to the frequencies in healthy donors. However, no significant differences were observed between moderate and severe patients (Fig. 1A). Moreover, the percentage of cycling memory but not effector CD8^+^ T cells subset significantly increased with disease severity (Fig. 1B). Furthermore, the results also showed that influenza patients had higher percentages of cycling CD4^+^ T cells than normal controls (Fig. 1C). In patients, cell cycling increased in CD8^+^ T cells subset in both CD27^high^ and CD27^low^ cells. The percentage of cycling memory CD4^+^ T cells subset increased with disease severity and there was no difference in the proportions of cycling effector CD4^+^ T cells between moderate and severe patients (Fig. 1D). Given that activated and expanded CD8^+^ T cells mainly enriched in the pool of Epstein-Barr viruses (EBV)-specific T cells during acute EBV infection [15], we have evaluated CD8^+^ T cells specific to influenza viruses. The response to the typical dominant epitope contributed to overall virus-specific CTL response[16]. Thus, these CD8^+^ T cells specific to the immunodominant M1_58-66_ epitope restricted by HLA-A*02:01 (A2/M1_58_) and PB1_498-505_ epitope restricted by HLA-A*24:02 (A24/PB1_498_) were involved in the analysis of results. Phenotypic analysis of these epitope-specific T cells revealed comparable cycling (Ki67^+^) and activated (CD38^+^HLA-DR^+^) phenotypes (Additional file 1: Fig. S1C). Moreover, the A2/M1_58_^+^CD8^+^ T cells and A24/PB1_498_^+^CD8^+^ T cells displayed an effector memory phenotype (TEM, substantially CD27^−^CD45RO^+^), which indicated that these epitope-specific T cells originated from an existing memory cells pool. Collectively, these data suggested that there was an increased memory cycling CD8^+^ and CD4^+^ T cells in severe patients during the acute phase of influenza infection. And these cell subsets partial share homology with influenza-specific T cells.

### The status of cycling T cells was associated with activation of T cells

**Fig. 2:**
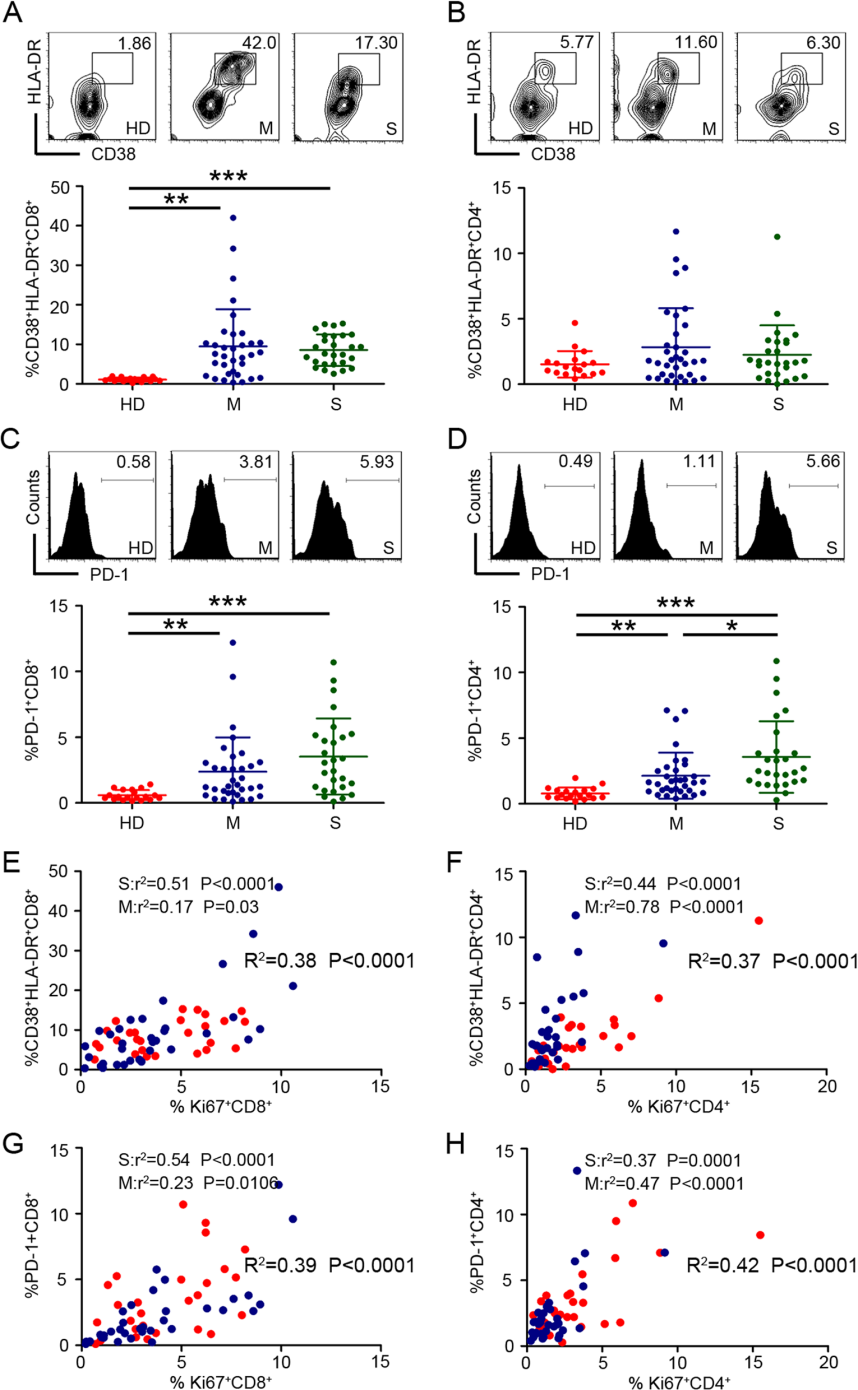
T cells of severe influenza patients highly express PD-1 but not CD38 and HLA-DR, and its expression was associated with that of Ki67. **A, B** Surface HLA-DR and CD38 expression among gated CD8^+^ and CD4^+^ T cells from healthy donors, moderate and severe influenza patients. **C, D** Surface PD-1 expression among gated CD8^+^ and CD4^+^ T cells from healthy donors and influenza patients. For each phenotype, the representative FACS plots (in top) and observed expression levels (in bottom) are shown separately. **E, F** Relationship between the percentage of Ki67^+^ T cells and CD38^+^HLA-DR^+^ T cells for all CD8^+^ or CD4^+^ T cells in moderate and severe influenza patients. **G, H** Relationship between the percentage of Ki67^+^ T cells and PD-1^+^ T cells for all CD8^+^ or CD4^+^ T cells in influenza patients. Data are displayed as mean ± SEM. Statistical significance is determined by unpaired t test. *P < 0.05, **P < 0.01, ***P < 0.001

To further determine whether the cycling of CD8^+^ and CD4^+^ T cells was associated with immune signals of activation and co-inhibitory, we first assessed the activation (CD38^+^HLA-DR^+^ and PD-1^+^) status of these T cells. No significant differences were found in the CD38^+^HLA-DR^+^CD8^+^ T cells between moderate and severe cases during the acute phase (T1) of influenza infection. However, Influenza patients had higher frequencies of activated CD8^+^ T cells (Fig. 2A). These differences were not observed in the CD4^+^ T cells subset (Fig. 2B). The differences in PD-1^+^CD8^+^ T cells subset between patients and healthy donors were marked during the acute phase of influenza infection. Although there was no statistically significant difference, severe patients tended to have higher frequency of PD-1^+^CD8^+^ T cells than moderate patients (Fig. 2C). Obviously, the percentage of PD-1^+^CD4^+^ T cells subset increased with disease severity (Fig. 2D). The link among cycling and activation on CD8^+^ and CD4^+^ T cells was further assessed by examining the relationship between intracellular Ki67 and cellular expression of activation markers (CD38-HLA-DR), the relationship between Ki67 expression and PD-1 expression, respectively. We found an obvious correlation between Ki67 and CD38-HLA-DR expression, no matter in CD8^+^ or CD4^+^ T cells subset (Fig. 2E, F). Similarly, a clear correlation was also found between Ki67 and PD-1 expression (Fig. 2G, H). Of note, no significant correlation in moderate patients was found between intracellular Ki67 and main surface markers expression (CD38-HLA-DR and PD-1), despite integrated data from all influenza patients suggesting otherwise (Fig. 2E, G). Thus, these data demonstrated that cycling status of T cells were associated with the status of activation in patients during the acute phase of influenza infection.

### Cycling and activated CD8^+^ T cells subset are delayed in severe influenza patients

**Fig. 3:**
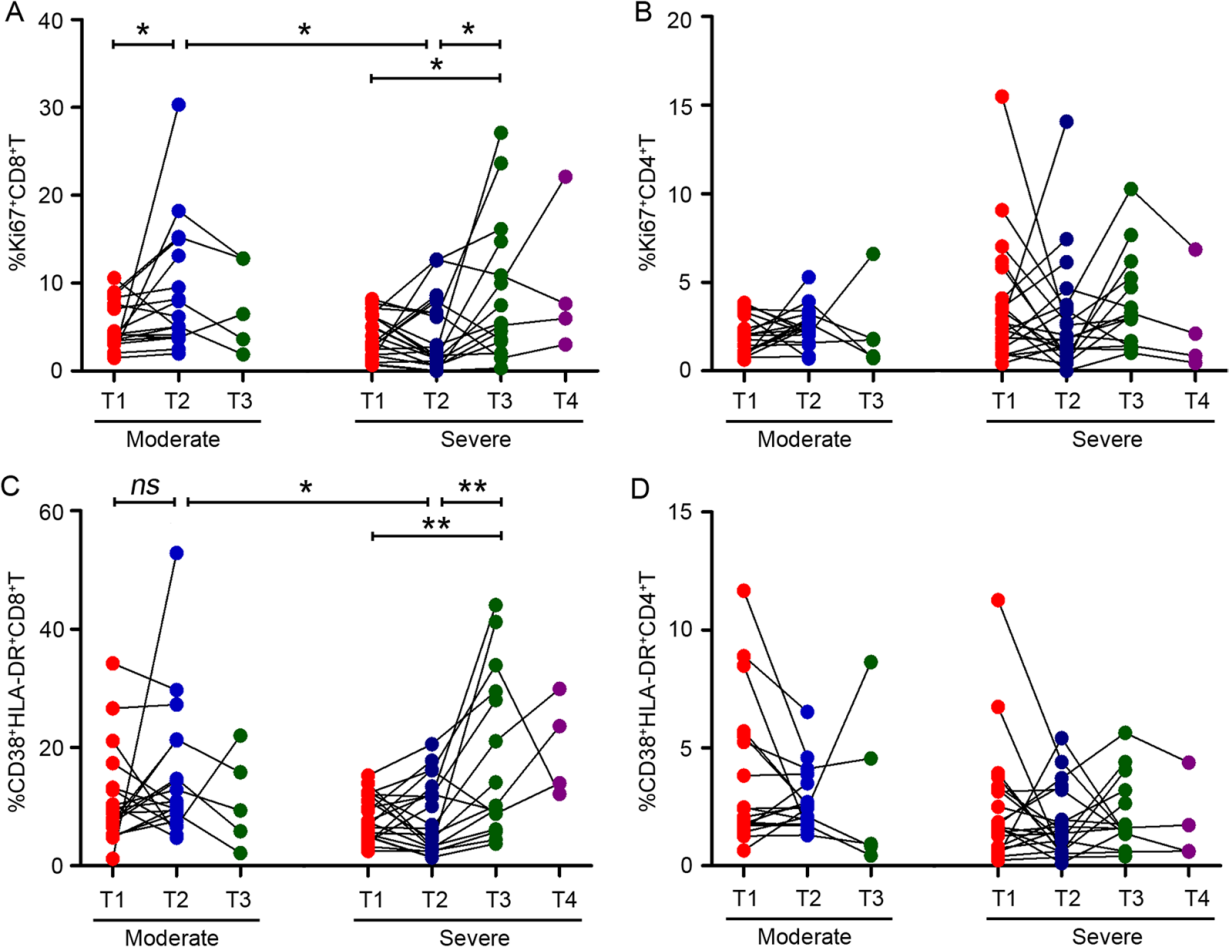
The kinetic changes of cycling and activated T cells during influenza infection were longitudinally characterized. Kinetics of cycling and activated T cells are shown as frequencies, **A** %Ki67^+^CD8^+^ T cells of total CD8^+^ T cells, **B** %Ki67^+^CD4^+^ T cells of total CD4^+^ T cells. **C** %CD38^+^HLA-DR^+^CD8^+^ T cells of total CD8^+^ T cells, **D** %CD38^+^HLA-DR^+^CD4^+^ T cells of total CD4^+^ T cells during the disease course. Statistical significance is determined by unpaired t test. *P < 0.05, **P < 0.01, *ns*, not significant

To characterize the kinetic changes of immune signals, longitudinally obtained PBMC cohort from 18 moderate and 21 severe influenza patients were analyzed. The frequency of Ki67^+^ T cells in influenza patients increased with the disease progression, especially at hospitalization (T1) to 3–5 days (T2) of moderate patients, at T1 to 8–10 days (T3) of severe patients. Strikingly, CD8^+^ T cells in moderate patients had high Ki67^+^ expression at T2, but in severe patients, CD8^+^ T cells had the same expression levels at T3 (Fig. 3A). Therefore, severe influenza patients displayed delayed kinetics of cycling (Ki67^+^) CD8^+^ T cells. In contrast, no significant difference was found in Ki67^+^CD4^+^ T cells between moderate and severe influenza patients (Fig. 3B). Furthermore, the frequency of activated (CD38^+^HLA-DR^+^) CD8^+^ T cells in severe influenza patients increased with the disease progression. However, in terms of the frequency of activated CD8^+^ T cells, although there was no statistically significant difference between T1 and T2 were observed in moderate patients, the frequency at T2 tended to be higher than that at T1 (Fig. 3C). Similarly, no significant difference was found in CD38^+^HLA-DR^+^CD4^+^ T cells between moderate and severe influenza patients (Fig. 3D). Collectively, these data suggested that early initiation of CD8^+^ T cells responses were associated with disease severity. Circulation of cycling and activated CD8^+^ T cells subset tended to delay in severe influenza patients.

## Discussion

The genes expression within cell cycle module displayed obvious differences between moderate and severe influenza infection [4]. Since the cell cycle has been widely believed to be required for cellular homeostasis [17], changes of several critical transition points in cell cycle pathways could be a clear immune signature for disease severity. Here, we investigated potential-immunophenotype in T cells of peripheral blood in patients hospitalized with moderate, severe seasonal influenza disease.

Compared with healthy controls, there was a larger proportion of cycling (Ki67^+^) T cells in the peripheral blood of influenza patients. However, no obvious difference was found in proportion of cycling CD8^+^ T cells between moderate and severe patients. The possible interpretation is that the priming, expansion and/or recall of T cells could be seen as a general and rapid stress response, and the order of response time and the response degree of CD8^+^ and CD4^+^ T cells could lead to indistinguishable in disease severity [18, 19]. We dissected the characteristics of cycling memory T cells in influenza patients and healthy donors. Our analysis indicated that the early elevation in proportion of cycling memory T cells had a certain predictive value for the severe progression of disease. A previous report evaluating peripheral blood samples found that the majority of activated CD8^+^ T cells (approximately 40%) were HIV-specific cells subset after acute HIV infection [20]. Similarly, we found that CD8^+^ T cells undergoing activation and division were primarily in the influenza virus-specific T cells subset, and highly expressed memory phenotype (CD45RO). These results suggested that the Ki67^+^CD38^+^HLA-DR^+^CD8^+^ T cells might be homologous with influenza virus-specific T cells subset. However, many of these Ki67^+^CD38^+^HLA-DR^+^ T cells subsets might not be IAV-specific, with this PBMC profile being associated with ineffective, early expansion of CD8 + T cell clones expressing TCRαβs known to recognize IAV epitopes in the patients[21]. Since most people had prior exposure to seasonal influenza, a high frequency of conserved peptides from influenza viruses-specific and cross-reactive CD8^+^ T lymphocytes might be pre-existing in the peripheral blood [22]. It seemed that the body had pre-formed memory cells pool from cytotoxic T lymphocyte (CTL), which could be quickly “awakened” after emerging seasonal influenza infection. These T cells originated from pool were rapidly activated and presented a divisive and proliferative state in an attempt to reduce viral load or alleviate clinical symptoms [23–25]. Certainly, it has been suggested that the effectiveness of this protective mechanism was affected by a variety of factors, including age, underlying disease, and whether exposed to influenza viral infection or not and the exposure duration [26–28].

Du et al. showed that HLA-DR^+^CD38^+^CD8^+ ^T cells were heterogeneous [29]. Similarly, in our study, a higher expression of HLA-DR^+^CD38^+^ was found in moderate influenza patients, implying activated immune responses or effective virus clearance, whereas lower expression of HLA-DR^+^CD38^+^ was found in the severe group, representing immune exhaustion or subsequently poor outcome. T cells exhaustion is a state of T cells with limited-function. It is defined by poor effector function, sustained expression of inhibitory receptors. Because pathogens or the induced inflammation still exist, the signal conduction of T cells gradually wanes and displays dysfunction or exhaustion. The exhausted T cells commonly express elevated inhibitory receptors like PD-1 or Tim-3 on the cell surface [30, 31]. Diao et al. showed that the surviving T cells in COVID-19 patients, particularly in those requiring ICU admission, appeared functionally exhausted [32]. Consistent with this report, here we found that both CD8^+^T cells and CD4^+^T cells had higher expression of PD-1 in influenza patients, particularly in the severe group during the acute phase of influenza infection. The possible explanation was prolonged viral shedding and long-lasting inflammatory response of severe influenza patients [33]. Therefore, the application of antiviral therapy combined with immunotherapy to limit the T cells exhaustion might be critical to severe influenza patients.

There were some limitations in our current study. First, the exact time of virus infection and the onset of symptoms were vague. For this reason, we only selected the enrollment time as the first time point. Second, due to the limited number of influenza patients, their grouping was heterogeneous in regard to underlying disease and the previous exposure history.

## Conclusions

Our present study proposed that delay in occurrence of cycling and activated T cells was associated with severe disease status. These findings provided new clues for identifying possible biological markers at the cellular level in peripheral blood to accurately predict the progression of influenza-induced disease severity.

## Methods

### Study design

Hospitalized patients were enrolled during the peak influenza season of 2017–2018 and 2019–2020 in the Department of Pulmonary and Critical Care Medicine from China-Japan Friendship Hospital. The study inclusion criteria required that subjects (i) were identified as laboratory-confirmed influenza infection using real-time PCR test; and (ii) had a time from onset of influenza-like symptoms (sore throat, fever of 38 ℃ or higher, cough) ≤ 14 days; and (iii) were able to obtain acute blood samples within 48 h after hospitalization. We excluded potential patients if they (i) were < 15 years old; (ii) pregnant; (iii) didn’t accept blood samples due to uncooperativeness. Peripheral blood of patients was obtained on admission (T1), 3–5 days (T2) and 8–10 days (T3) after enrollment. For some relatively serious influenza patients who had been in hospital for a long time (to week 4), blood samples were collected again 18–21 days later (T4). Disease severity was defined according to the degree of respiratory compromise and comprehensive clinical evaluation. Severe influenza was defined as patients with severe influenza pneumonitis and hypoxic respiratory failure that received invasive mechanical ventilation and/or emergency extracorporeal membrane oxygenation (ECMO); Moderate influenza was defined as patients with supplemental oxygen therapy (including respiratory compromise requiring nasal high-flow oxygen therapy and/or noninvasive mechanical ventilatory support) or not. Healthy individuals enrolled had not experienced influenza-like symptoms or vaccinated for influenza within the three months. Peripheral blood mononuclear cells (PBMCs) were isolated from whole blood samples and stored in liquid nitrogen until using (Additional file 1: Fig. S1A).

### Flow cytometric analysis

The PBMCs were thawed and analyzed using a panel of antibodies as follows: PE-eFluor 610 anti-human Ki67 (Thermo/eBio); FITC anti-human CD8α (clone Hit8α), PE anti-human CD4 (clone RPA-T4), PE-Cyanine7 anti-human CD38 (clone HB7), violetFluor™450 anti-human HLA-DR (clone LN3), APC-Cyanine7 anti-human CD27 (clone O323), APC anti-human CD279 (PD-1) (clone J110), PerCP-Cyanine5.5 anti-human CD45RO (clone UCHL1), redFluor™ 710 anti-human CD45RA (clone HI100) (TONBO biosciences). The cell number was generally at least 5 × 10^6^ per tube. The PBMCs were washed twice and resuspended in 100 μl of phosphate-buffered saline (PBS), and then stained with PI for the cell viability testing. And then stained in flow cytometry staining buffer with appropriate diluted concentrations of the antibodies, then fixed, permeated and intracellularly stained with anti-human Ki67 (Thermo/eBio) using the Foxp3/Transcription factor staining kits (TONBO biosciences). Cells were resuspended and acquired on a CytExpert software (Beckman Coulter) and analyzed by FlowJo software (Treestar). The frequency of epitope-specific CD8^+^ T cells and their main immunophenotypes was measured by staining cells with surface markers CD8α, CD3, CD38, HLA-DR, CD27, CD45RO (TONBO biosciences) and intracellular marker Ki67 (Thermo/eBio), with T-select MHC Tetramer (M1_58_, HLA-A*02:01-GILGFVFTL)-PE or (PB1_498_, HLA-A*24:02-RYGFVANF) (MBL, Beijing Biotech) on 24 h of the culture and then analyzed by flow cytometry.

### HLA type, peptide and tetramers

To conduct MHC-tetramer staining in PBMC, HLA typing was required. HLA typing was performed by multiplex PCR of HLA specific amplicons. HLA sequences were tagged with biotin-labeled DNA probe, then efficiently pooled and sequenced. Influenza A peptides derived from M1 and PB1 proteins and formed MHC tetramer were shown in Additional file 2: Fig. S2.

### Statistical analysis

Statistical analysis was performed using Graph-Pad Prism 5.0 software and statistical significance was calculated by Student's t test, Mann–Whitney U test, χ2 test, or Fisher's exact test, as appropriate. *P < 0.05, **P < 0.01 and ***P < 0.001 represented significant differences.

## Supplementary Information

**Additional file 1: **Fig S1. Study design overview and representative FACS plots.

**Additional file 2: **Fig S2. Highly conserved influenza epitopes used in the study.

## Data Availability

The datasets used and/or analyzed during the current study available from the corresponding author on reasonable request.

## Abbreviations

ECMO: Extra corporeal membrane oxygenation
PBMCs: Peripheral blood mononuclear cells
HLA: Human leukocyte antigen
EBV: Epstein-Barr viruses
CTL: Cytotoxic T lymphocyte
FACS: Fluorescence activating cell sorter
