# Supplementary material for: Cycling and activated CD8+ T lymphocytes and their association with disease severity in influenza patients
Source: BMC Immunol. 2022 Sep 5;23:40. doi: 10.1186/s12865-022-00516-1 (PMC9441835; doi:10.1186/s12865-022-00516-1)
Supplement: Supplementary file 1 — Additional file 1: Fig S1. Study design overview and representative FACS plots. [file 12865_2022_516_MOESM1_ESM.pdf]

Fig. S1

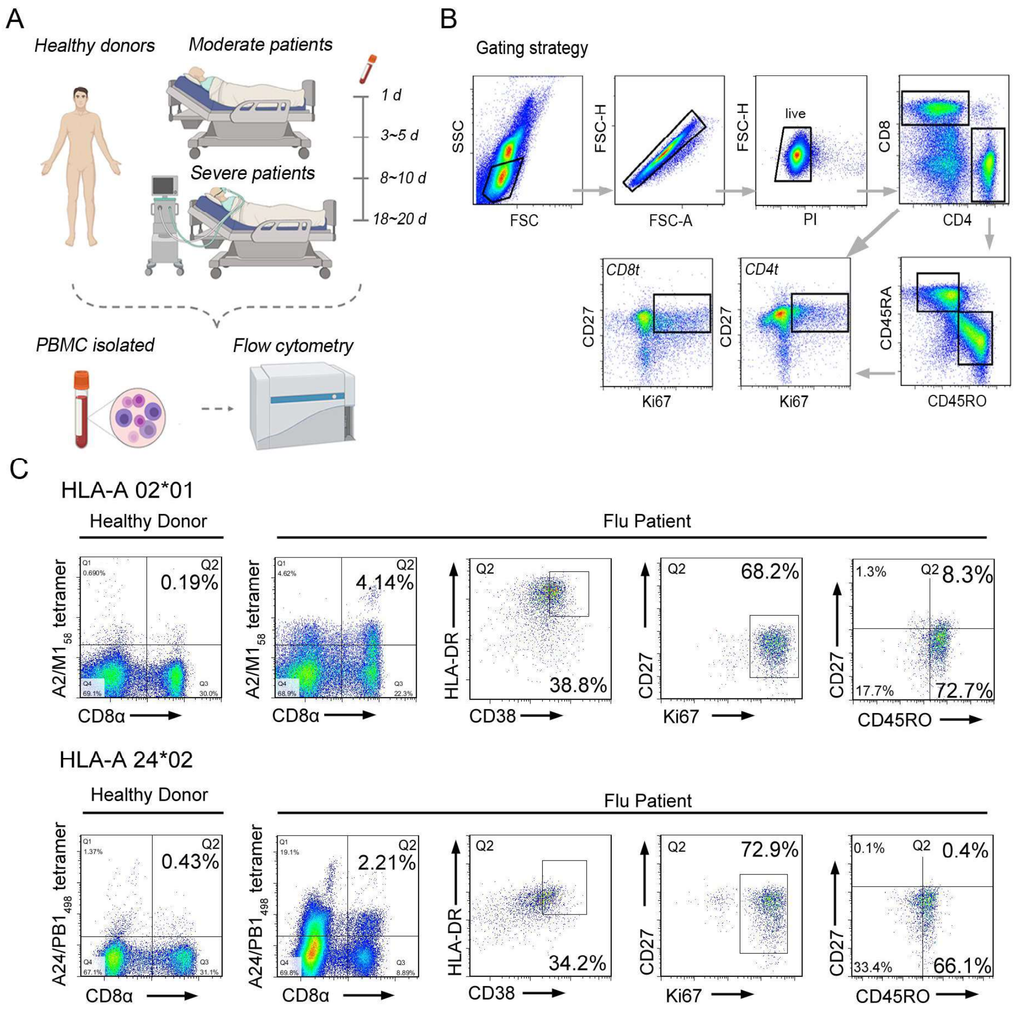

Fig. S1. Study design and representative FACS plots. A. Study design overview (created in BioRender.com). **B.** Representative FACS plots displaying surface staining of CD8, CD4, CD45RO, CD45RA and CD27; and intracellular Ki67 staining in PBMC from influenza patients. **C.** Representative FACS plots of A2/M1$_{58}$$^{+}$CD8^{+}, A24/PB1$_{498}$$^{+}$CD8^{+} T cells and their main immunological phenotype from enriched samples of influenza patients PBMCs.
