# Supplementary material for: Cycling and activated CD8+ T lymphocytes and their association with disease severity in influenza patients
Source: BMC Immunol. 2022 Sep 5;23:40. doi: 10.1186/s12865-022-00516-1 (PMC9441835; doi:10.1186/s12865-022-00516-1)
Supplement: Supplementary file 2 — Additional file 2: Fig S2. Highly conserved influenza epitopes used in the study. [file 12865_2022_516_MOESM2_ESM.pdf]

Fig. S2

HLA restriction	Peptide	Sequence	Epitope acronym
**HLA-A*02:01**	$M1_{58\text{-}66}$	GILGFVFTL	$A2/M1_{58}$
**HLA-A*24:02**	$PB1_{498\text{-}505}$	RYGFVANF	$A24/PB1_{498}$

Fig.S2. Highly conserved influenza epitopes used in the study.
